# 4,4′-Diphenyl-2,2′-bi-1,3-thia­zole

**DOI:** 10.1107/S1600536810027261

**Published:** 2010-07-17

**Authors:** Seik Weng Ng

**Affiliations:** a Department of Chemistry, University of Malaya, 50603 Kuala Lumpur, Malaysia

## Abstract

In the centrosymmetric title compound, C_18_H_12_N_2_S_24_, the five- (r.m.s. deviation = 0.002 Å) and six-membered (r.m.s. deviation = 0.002 Å) rings are essentially coplanar [dihedral angle between rings = 1.9 (1)°].

## Related literature

For the crystal structures of other 4,4′-disubstituted compounds, see: Bolognesi *et al.* (1987 ▶); Craig *et al.* (1988 ▶); Curtis *et al.* (2004 ▶).
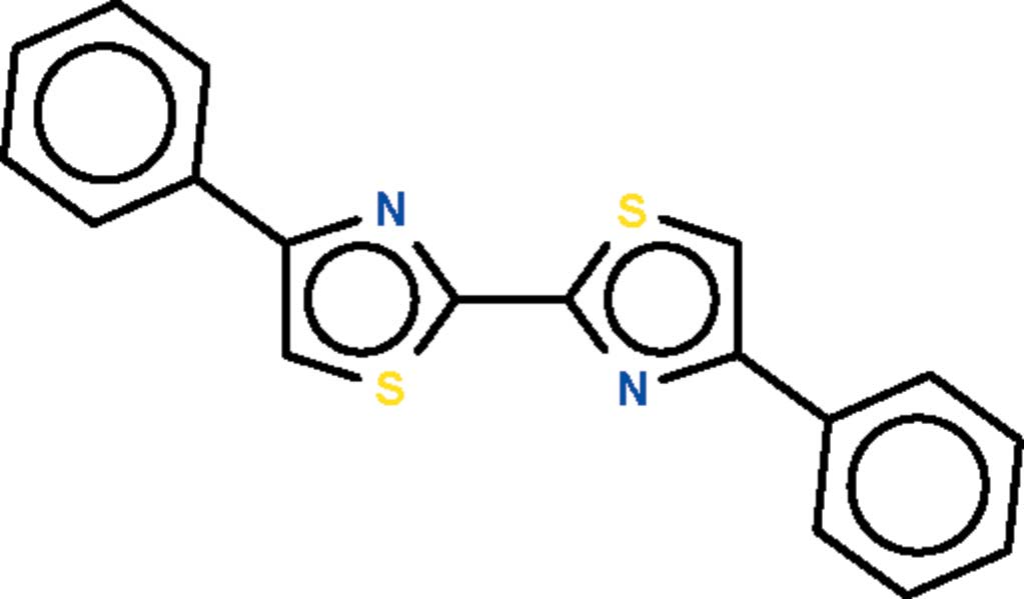


## Experimental

### 

#### Crystal data

C_18_H_12_N_2_S_2_
*M* *_r_* = 320.42
Monoclinic, 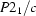

*a* = 5.7769 (4) Å
*b* = 7.6573 (5) Å
*c* = 17.1960 (12) Å
β = 99.614 (1)°
*V* = 749.99 (9) Å^3^
*Z* = 2
Mo *K*α radiation
μ = 0.35 mm^−1^
*T* = 100 K
0.30 × 0.10 × 0.10 mm

#### Data collection

Bruker SMART APEX diffractometer
Absorption correction: multi-scan (*SADABS*; Sheldrick, 1996 ▶) *T*_min_ = 0.902, *T*_max_ = 0.966
6993 measured reflections
1730 independent reflections
1575 reflections with *I* > 2σ(*I*)
*R*_int_ = 0.026

#### Refinement

*R*[*F* ^2^ > 2σ(*F* ^2^)] = 0.028
*wR*(*F* ^2^) = 0.079
*S* = 1.04
1730 reflections
100 parameters
H-atom parameters constrained
Δρ_max_ = 0.42 e Å^−3^
Δρ_min_ = −0.24 e Å^−3^

### 

Data collection: *APEX2* (Bruker, 2009 ▶); cell refinement: *SAINT* (Bruker, 2009 ▶); data reduction: *SAINT*; program(s) used to solve structure: *SHELXS97* (Sheldrick, 2008 ▶); program(s) used to refine structure: *SHELXL97* (Sheldrick, 2008 ▶); molecular graphics: *X-SEED* (Barbour, 2001 ▶); software used to prepare material for publication: *publCIF* (Westrip, 2010 ▶).

## Supplementary Material

Crystal structure: contains datablocks global, I. DOI: 10.1107/S1600536810027261/nk2046sup1.cif

Structure factors: contains datablocks I. DOI: 10.1107/S1600536810027261/nk2046Isup2.hkl

Additional supplementary materials:  crystallographic information; 3D view; checkCIF report

## Figures and Tables

**Fig. 1.**
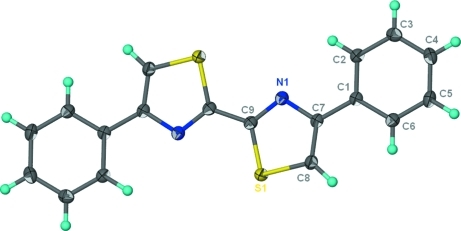
Displacement ellipsoid plot (Barbour, 2001) of C18H12N2S2 at the 70% probability level. Hydrogen atoms are drawn as spheres of arbitrary radius. The molecule lies on an inversion center.

## supplementary crystallographic information

### Comment

2,2'-Bithiazole and other 4,4'-disubstituted derivatives possess a pair of nitrogen-donor sites that renders such molecules capable of chelating metal atoms. The crystal structure of the parent compound as well as those of the methyl and ethyl substituted derivatives have been reported (Bolognesi *et al.*, 1987; Craig *et al.*, 1988, Curtis *et al.*, 2004). These molecules are centrosymmetric compounds having an inversion center midway along the C_azolyl_–C_azolyl_ bond. In the parent compound, this bond is 1.468 (6) Å (Bolognesi *et al.*, 1987). The bond is somewhat shortened to 1.455 (2) Å in the phenyl analog (Scheme I, Fig. 1).

### Experimental

The organic compound was returned unchanged in an attempted reaction of lead(II) nitrate (0.13 mmol, 0.04 g) with 4,4'-diphenyl-2,2'-bithiazole (0.25 mmol, 0.08 g) in the presence of potassium thiocyanate (0.25 mmol, 0.03 g) in a methanol/THF mixture. Crystals were obtained after one week of setting the mixture aside.

### Refinement

Hydrogen atoms were placed in calculated positions (C–H 0.95 Å) and included in the refinement in the riding model approximation, with *U*(H) set to 1.2*U*_eq_(C).

### Crystal data

**Table:**

C_18_H_12_N_2_S_2_	*F*(000) = 332
*M**_r_* = 320.42	*D*_x_ = 1.419 Mg m^−^^3^
Monoclinic, *P*2_1_/*c*	Mo *K*α radiation, λ = 0.71073 Å
Hall symbol: -P 2ybc	Cell parameters from 4091 reflections
*a* = 5.7769 (4) Å	θ = 2.4–28.3°
*b* = 7.6573 (5) Å	µ = 0.35 mm^−^^1^
*c* = 17.1960 (12) Å	*T* = 100 K
β = 99.614 (1)°	Prism, colorless
*V* = 749.99 (9) Å^3^	0.30 × 0.10 × 0.10 mm
*Z* = 2	

### Data collection

**Table:**

Bruker SMART APEX diffractometer	1730 independent reflections
Radiation source: fine-focus sealed tube	1575 reflections with *I* > 2σ(*I*)
graphite	*R*_int_ = 0.026
ω scans	θ_max_ = 27.5°, θ_min_ = 2.4°
Absorption correction: multi-scan (*SADABS*; Sheldrick, 1996)	*h* = −7→7
*T*_min_ = 0.902, *T*_max_ = 0.966	*k* = −9→9
6993 measured reflections	*l* = −21→22

### Refinement

**Table:**

Refinement on *F*^2^	Primary atom site location: structure-invariant direct methods
Least-squares matrix: full	Secondary atom site location: difference Fourier map
*R*[*F*^2^ > 2σ(*F*^2^)] = 0.028	Hydrogen site location: inferred from neighbouring sites
*wR*(*F*^2^) = 0.079	H-atom parameters constrained
*S* = 1.04	*w* = 1/[σ^2^(*F*_o_^2^) + (0.0421*P*)^2^ + 0.350*P*] where *P* = (*F*_o_^2^ + 2*F*_c_^2^)/3
1730 reflections	(Δ/σ)_max_ = 0.001
100 parameters	Δρ_max_ = 0.42 e Å^−^^3^
0 restraints	Δρ_min_ = −0.24 e Å^−^^3^

### Fractional atomic coordinates and isotropic or equivalent isotropic displacement parameters (Å^2^)

**Table:**

	*x*	*y*	*z*	*U*_iso_*/*U*_eq_	
S1	0.76174 (5)	0.35670 (4)	0.446214 (17)	0.01797 (12)	
N1	0.35546 (18)	0.49301 (13)	0.39798 (6)	0.0151 (2)	
C1	0.2750 (2)	0.43006 (15)	0.25545 (7)	0.0139 (2)	
C2	0.0586 (2)	0.51551 (16)	0.24862 (7)	0.0156 (2)	
H2	0.0132	0.5690	0.2936	0.019*	
C3	−0.0906 (2)	0.52260 (16)	0.17611 (7)	0.0179 (3)	
H3	−0.2377	0.5804	0.1719	0.022*	
C4	−0.0255 (2)	0.44574 (17)	0.10999 (7)	0.0198 (3)	
H4	−0.1278	0.4505	0.0607	0.024*	
C5	0.1903 (2)	0.36157 (16)	0.11614 (7)	0.0193 (3)	
H5	0.2355	0.3094	0.0708	0.023*	
C6	0.3399 (2)	0.35345 (15)	0.18827 (7)	0.0165 (3)	
H6	0.4868	0.2957	0.1921	0.020*	
C7	0.4293 (2)	0.41960 (15)	0.33292 (7)	0.0140 (2)	
C8	0.6444 (2)	0.34026 (16)	0.34867 (7)	0.0167 (3)	
H8	0.7186	0.2835	0.3103	0.020*	
C9	0.5133 (2)	0.46895 (15)	0.46100 (7)	0.0150 (2)	

### Atomic displacement parameters (Å^2^)

**Table:**

	*U*^11^	*U*^22^	*U*^33^	*U*^12^	*U*^13^	*U*^23^
S1	0.01731 (17)	0.02092 (18)	0.01515 (17)	0.00472 (11)	0.00112 (12)	−0.00100 (11)
N1	0.0165 (5)	0.0146 (5)	0.0145 (5)	−0.0003 (4)	0.0035 (4)	0.0003 (4)
C1	0.0159 (6)	0.0113 (5)	0.0149 (6)	−0.0023 (4)	0.0038 (4)	0.0007 (4)
C2	0.0166 (6)	0.0142 (6)	0.0167 (6)	−0.0010 (4)	0.0046 (5)	−0.0004 (4)
C3	0.0156 (6)	0.0155 (6)	0.0221 (6)	−0.0006 (4)	0.0014 (5)	0.0004 (5)
C4	0.0229 (7)	0.0179 (6)	0.0167 (6)	−0.0023 (5)	−0.0020 (5)	−0.0003 (5)
C5	0.0256 (7)	0.0179 (6)	0.0147 (6)	−0.0011 (5)	0.0044 (5)	−0.0030 (4)
C6	0.0180 (6)	0.0149 (6)	0.0172 (6)	0.0006 (4)	0.0040 (5)	−0.0007 (4)
C7	0.0168 (6)	0.0115 (5)	0.0145 (5)	−0.0017 (4)	0.0043 (4)	−0.0004 (4)
C8	0.0187 (6)	0.0176 (6)	0.0141 (5)	0.0006 (4)	0.0034 (4)	−0.0013 (4)
C9	0.0164 (6)	0.0134 (5)	0.0157 (6)	0.0001 (4)	0.0041 (4)	0.0001 (4)

### Geometric parameters (Å, °)

**Table:**

S1—C8	1.7056 (12)	C3—H3	0.9500
S1—C9	1.7278 (12)	C4—C5	1.3914 (18)
N1—C9	1.3076 (15)	C4—H4	0.9500
N1—C7	1.3817 (15)	C5—C6	1.3897 (17)
C1—C2	1.3981 (16)	C5—H5	0.9500
C1—C6	1.4013 (16)	C6—H6	0.9500
C1—C7	1.4762 (16)	C7—C8	1.3690 (17)
C2—C3	1.3934 (17)	C8—H8	0.9500
C2—H2	0.9500	C9—C9^i^	1.455 (2)
C3—C4	1.3870 (18)		
			
C8—S1—C9	88.74 (6)	C6—C5—H5	119.9
C9—N1—C7	110.30 (10)	C4—C5—H5	119.9
C2—C1—C6	119.02 (11)	C5—C6—C1	120.33 (11)
C2—C1—C7	119.86 (11)	C5—C6—H6	119.8
C6—C1—C7	121.11 (11)	C1—C6—H6	119.8
C3—C2—C1	120.28 (11)	C8—C7—N1	114.40 (11)
C3—C2—H2	119.9	C8—C7—C1	126.45 (11)
C1—C2—H2	119.9	N1—C7—C1	119.14 (10)
C4—C3—C2	120.33 (11)	C7—C8—S1	111.12 (9)
C4—C3—H3	119.8	C7—C8—H8	124.4
C2—C3—H3	119.8	S1—C8—H8	124.4
C3—C4—C5	119.76 (11)	N1—C9—C9^i^	123.47 (14)
C3—C4—H4	120.1	N1—C9—S1	115.44 (9)
C5—C4—H4	120.1	C9^i^—C9—S1	121.09 (12)
C6—C5—C4	120.27 (11)		
			
C6—C1—C2—C3	0.60 (17)	C6—C1—C7—C8	0.67 (18)
C7—C1—C2—C3	−178.53 (11)	C2—C1—C7—N1	0.83 (16)
C1—C2—C3—C4	−0.32 (18)	C6—C1—C7—N1	−178.29 (11)
C2—C3—C4—C5	−0.17 (18)	N1—C7—C8—S1	−0.35 (13)
C3—C4—C5—C6	0.36 (19)	C1—C7—C8—S1	−179.35 (9)
C4—C5—C6—C1	−0.06 (18)	C9—S1—C8—C7	0.34 (10)
C2—C1—C6—C5	−0.41 (17)	C7—N1—C9—C9^i^	−179.94 (14)
C7—C1—C6—C5	178.71 (11)	C7—N1—C9—S1	0.14 (13)
C9—N1—C7—C8	0.13 (15)	C8—S1—C9—N1	−0.28 (10)
C9—N1—C7—C1	179.22 (10)	C8—S1—C9—C9^i^	179.80 (14)
C2—C1—C7—C8	179.79 (12)		

Symmetry codes: (i) −*x*+1, −*y*+1, −*z*+1.
